# 妊娠期铁缺乏及缺铁性贫血管理新进展

**DOI:** 10.3760/cma.j.cn121090-20250826-00402

**Published:** 2026-04

**Authors:** 娟 张, 苗 陈

**Affiliations:** 1 中国医学科学院北京协和医院血液内科，北京 100730 Department of Hematology, Peking Union Medical College Hospital and Chinese Academy of Medical Sciences, Beijing 100730, China; 2 北京市第六医院血液内科，北京 100007 Department of Hematology, Beijing Sixth Hospital, Beijing 100007, China

## Abstract

妊娠期铁缺乏及缺铁性贫血（iron deficiency anemia, IDA）是常见妊娠并发症，可导致多种不良妊娠结局。本文基于最新循证医学证据综述妊娠期铁缺乏及IDA的管理策略。对于口服铁剂不耐受、疗效不佳或需快速纠正贫血的中重度IDA患者，静脉铁剂治疗具有显著优势。新一代静脉铁剂提高了治疗效率，但其在孕早期的安全性仍需进一步评估，使用时需监测低磷血症等不良反应。本文还分析了静脉铁剂相关过敏反应与补体激活相关假性过敏反应的鉴别诊断和处理流程。临床实践中应根据患者具体情况个体化选择治疗方案，遵循循证医学指南，优化母婴结局。

## Abstract (en)

Iron deficiency（ID）and iron deficiency anemia（IDA）are common obstetric complications associated with various adverse pregnancy outcomes. According to the latest evidence-based medical evidence, this article reviews the management strategies for ID and IDA during pregnancy. Intravenous iron therapy provides substantial advantages for patients who cannot tolerate or do not respond to oral iron, as well as for those with severe IDA requiring rapid correction. The application of new-generation intravenous iron agents has improved treatment efficiency; however, their safety in the first trimester warrants further evaluation. Further, monitoring for adverse events, including hypophosphatemia, is necessary. This review further examines the differential diagnosis and management of intravenous iron-related hypersensitivity reactions and complement activation-related pseudoallergy. Management should be individualized based on patient-specific factors in clinical practice, with adherence to evidence-based guidelines to optimize maternal and infant outcomes.

缺铁性贫血（IDA）是全球最常见的营养缺乏性疾病，在妊娠期成为一个重要的临床问题。IDA不仅与子痫前期、产后出血等母体并发症密切相关，还显著增加胎儿生长受限、早产、低出生体重等不良围产结局发生率。近年研究还揭示母体铁代谢异常对子代神经发育的深远影响，妊娠期铁缺乏可干扰胎儿脑发育，增加后代认知功能障碍、运动协调异常及神经精神疾病风险[1]。且由于母乳中铁生物利用度较低，胎儿期铁储备不足还将导致新生儿持续性铁缺乏。WHO建议妊娠期每日补充30～60 mg元素铁，可有效降低低出生体重发生率[2]。

尽管国际指南已明确妊娠期IDA的规范化诊疗路径，临床实践中仍存在管理缺口。我国孕产妇静脉铁剂使用率远低于欧美国家，主要源于对静脉铁剂潜在风险的担忧、门诊输注条件不足及治疗成本较高等因素。近年多项随机对照试验（RCT）证实，对于中重度IDA或口服铁剂不耐受的孕妇，静脉铁剂可快速纠正贫血且未增加不良妊娠结局。本文系统梳理最新循证证据，重点探讨静脉铁剂在妊娠各期的安全性特征，以推动我国围产期铁缺乏管理策略的优化。

一、妊娠期铁缺乏的危害

1. 流行病学特征：流行病学数据显示，妊娠期IDA总体患病率超过30％，妊娠晚期孕妇缺铁的发生率高达84％[3]。我国多中心研究（12 403例）显示，妊娠期贫血总体患病率为19.8％，其中IDA占比达70.2％，且发病率随孕周增加而升高，至妊娠32周达到峰值[4]。鉴于铁储备耗竭的发生率显著高于IDA，亚临床铁缺乏的潜在危害可能被严重低估。

2. IDA相关母胎风险：母体层面：除典型贫血引起的呼吸困难、疲劳、运动耐力下降、心悸和睡眠障碍等外，IDA可引发先兆子痫、子痫、胎盘早剥、产后抑郁和围产期输血需求增加。胎儿层面：IDA增加早产、小于胎龄儿或低出生体重及新生儿死亡风险。Sowe等[5]发现母亲贫血与儿童贫血之间存在显著代际关联，母亲贫血的儿童，患贫血的可能性比母亲不贫血的儿童高出13.5％。2022年一项基于中国18 948 443名孕妇（包括3 369 653名贫血孕妇和15 578 790名非贫血孕妇）的回顾性队列研究表明，重度贫血（HGB<70 g/L）孕妇发生胎儿生长受限的风险比正常孕妇高约8％[6]。母亲患有IDA的新生儿脐带血铁蛋白浓度较非IDA组降低约16.5％[7]。

3. 对胎儿发育的影响：铁元素是中枢神经系统发育的关键元素，妊娠期铁缺乏可能导致神经元功能障碍、髓鞘异常及突触紊乱，引起认知发育迟缓、自闭症谱系障碍及注意缺陷多动障碍[8]。从受孕至出生后24个月是大脑发育的关键发育窗口期，此阶段发生铁缺乏，即使后期补铁也难以完全逆转脑发育缺陷[9]。

Gundacker等[10]报道，小鼠孕期及哺乳期轻度缺铁，尽管幼鼠在补铁后脑铁水平恢复正常，但成年后仍表现出显著的抑郁和焦虑样行为，且海马miRNA表达谱发生持久改变。该研究为理解早期缺铁与成年后情绪障碍之间的关系提供了重要的依据。Hua等[11]研究发现，胎儿期铁缺乏儿童完成任务的整体准确率明显低于铁充足组，且大脑激活模式存在差异，提示早期铁缺乏对认知控制功能产生长期影响。

机制研究方面，IDA可通过多重机制损伤神经血管单元结构与功能。Isasi等[12]发现，大鼠孕期缺铁会导致后代大脑胼胝体中的神经纤维髓鞘化不足、星形胶质细胞不成熟、血脑屏障完整性破坏及周细胞数量减少或结构异常，加剧脑组织缺血缺氧状态，引发神经系统损伤。

铁元素还参与多种氧化还原酶的催化反应，对维持细胞代谢和胚胎器官发育至关重要。孕期铁缺乏干扰这些酶的活性，从而影响胎儿器官的正常形成与功能。英国一项病例对照研究发现，孕早期贫血母亲生下先天性心脏病胎儿的概率高出47％[13]。Agaoglu等[14]的前瞻性研究显示，IDA孕妇的胎儿心脏收缩和舒张功能显著低于健康对照组。动物实验发现宫内铁缺乏可导致大鼠肾单位数量减少、肾小球生成周期延长，并诱发肾素-血管紧张素系统异常激活[15]。这些结构功能改变与成年期高血压及肾功能减退密切相关，印证了发育源性疾病的“胎源假说”，即胎儿期营养环境不良可编程成年后慢性疾病的发生风险[16]。

2025年《Nature》杂志发表的一项突破性成果证实，铁代谢在小鼠雄性性别决定中起到关键作用。母体膳食铁缺乏可导致部分子代出现雄性向雌性的性腺性别逆转，揭示了母体铁水平与胎儿发育结局的关联机制[17]。这些发现强调了母体在妊娠期维持足够铁水平的重要性。

二、妊娠期铁缺乏及IDA的诊断与治疗

妊娠期铁缺乏的筛查通常包括3个关键时间点：首次产前检查、妊娠晚期及分娩入院时。WHO将妊娠期贫血定义为HGB<110 g/L，将妊娠早期铁缺乏定义为铁蛋白<15 µg/L[18]。当血清铁蛋白<30 µg/L或转铁蛋白饱和度<20％时，提示体内铁储备耗竭[19]。建议及时启动干预。

1. 口服铁剂治疗：确诊后尽早启动口服铁剂治疗。与常规每日补铁（60 mg/d）相比，间歇性补铁方案（120 mg/d，隔日1次）可更显著提升HGB水平，同时减少胃肠道不良反应[20]。补铁2～4周后复查HGB，有效应答标准为HGB上升≥10 g/L。HGB恢复正常后应持续治疗至少3个月或至产后6周。

2. 静脉铁剂治疗：应用指征[21]：①口服铁剂不耐受或疗效欠佳；②中重度贫血（HGB≤90 g/L）；③临近分娩需快速纠正贫血。围产期静脉补铁在提升HGB水平方面具有优势。一项Meta分析（13项RCT，3 939例）显示，静脉补铁组HGB平均值比口服补铁组高4.9 g/L（治疗3～6周后），分娩时平均提升5.5 g/L[22]。另一项Meta分析（33项RCT，4 558例）显示，针对产后6周内HGB≤120 g/L的产妇，静脉补铁具有改善早期疲劳症状的优势[23]。

静脉铁剂历经三代演化：第一代制剂高分子右旋糖酐铁及胶体氢氧化铁，因严重过敏反应风险已退出临床；第二代制剂蔗糖铁、低分子右旋糖酐铁及葡萄糖酸铁，过敏风险显著降低，但其稳定性限制单次剂量，需分次输注；第三代制剂羧基麦芽糖铁（ferric carboxymaltose，FCM）、异麦芽糖酐铁（Ferric derisomaltose，FDI）及纳米氧化铁（ferumoxytol），以Fe（III）-羟基氧化物铁核共价结合多糖配体，结构更稳定，降低了游离铁相关氧化应激风险[24]。动物实验及药代动力学研究显示FCM经胎盘转运及乳汁分泌的暴露水平较低[25]，在妊娠期及哺乳期患者中具有应用价值。一项真实世界研究（1 191例孕妇）显示，FCM单次输注后4周HGB平均提升28 g/L；药物相关不良事件发生率为8.6％，主要为轻度消化道症状（恶心5.0％，便秘0.2％）及头痛（3.7％）[26]。2024年一项开放标签RCT研究（1 056例）显示，FCM单次输注较口服铁剂HGB改善更快[27]。一项Meta分析（9项RCT，1 406例）显示，FCM较蔗糖铁可多提升HGB 8.9 g/L，且输注效率提高10倍（15 min对3～5 h）[28]。2025年一项多中心RCT（4 368例）显示，静脉FCM和FDI组贫血纠正达标率均高于口服铁剂组，FCM组的低出生体重发生率低于口服铁剂组[29]。

临床决策建议：对于血流动力学稳定且无急性失血症状的孕产妇，推荐阶梯式治疗策略——首选口服铁剂，若治疗2周应答不足（HGB增幅<10 g/L）或存在不耐受，或因妊娠晚期、产后等情况需快速补铁，则升级为静脉补铁[30]。

三、铁剂治疗的安全性

1. 口服铁剂的不良反应：主要表现为剂量依赖性胃肠道反应，包括恶心、腹痛、便秘及黑便。妊娠期患者因孕酮水平升高导致胃肠动力减弱，耐受性进一步下降。即便采用间歇性补铁方案，仍有18％～25％的孕妇因不良反应调整治疗[31]。

2. 静脉铁剂的不良反应：

（1）氧化应激风险：妊娠早期（<12周）使用静脉铁剂仍存争议。铁在胎盘中含量丰富，具有通过产生活性氧（ROS）诱导氧化应激的潜力。动物实验显示，铁过载与炎症共同作用时可显著增加胚胎丢失和畸形率[32]。鉴于孕早期应用静脉铁剂的安全性尚未完全明确，国内外学者建议在孕中期或孕晚期进行静脉补铁[33]–[34]。

（2）过敏反应和Fishbane反应：静脉铁剂输注可能引发过敏反应，表现为呼吸困难、低血压、皮疹、瘙痒等，严重时可出现休克。美国食品药品监督管理局（FDA）不良事件报告系统数据库显示，纳米氧化铁和右旋糖酐铁过敏反应及过敏性休克报告率最高，不同静脉铁剂过敏反应的发生率和严重程度差异显著[35]。欧洲一项大规模研究（304 210例次）显示，首次使用静脉注射铁剂的过敏性休克发生率为0.4～0.5/10 000次给药；第二次使用时过敏性休克的发生率为0.25/10 000次给药，第三次及以后使用时为0.02/10 000次给药[36]。瑞典一项回顾性研究中，213例患者FDI治疗的轻度过敏反应发生率4.7％，均为一过性，未见严重不良事件[37]。

Fishbane反应属于补体激活相关假性过敏反应（CARPA），由静脉铁剂纳米颗粒直接激活补体系统，生成C3a、C5a等过敏毒素，刺激肥大细胞和嗜碱性粒细胞释放组胺、类胰蛋白酶等介质，导致血管扩张、毛细血管通透性增加及平滑肌收缩，出现类似过敏反应的表现。该反应通常发生在输注开始后数分钟至1 h内，表现为潮红、瘙痒、皮疹、胸部压迫感、背痛等，严重者可出现低血压、心动过速、呼吸困难。减慢输注速率或暂停输注后症状迅速缓解，呈自限性，一般无需使用肾上腺素或抗组胺药物治疗[38]。2024年美国胃肠病学会专家综述指出，IgE介导的真正过敏反应极为少见，CARPA是更为常见的机制[39]。

过敏反应和Fishbane反应在临床表现上存在重叠，但两者在发病机制、处理策略及预后方面具有本质差异。基于现有文献，图1对两种反应的病理生理机制、临床特征及处理流程进行了归纳。静脉铁剂输注应在具备过敏反应识别与处理能力的医疗机构中进行。输注前应告知患者可能出现的反应，一旦发生应立即停止输注并评估严重程度，必要时给予抗组胺药和糖皮质激素治疗。严重过敏反应需予肾上腺素、补液、高流量吸氧等紧急处理[40]。抗组胺药物西替利嗪和氯雷他定在妊娠期使用时未显著增加不良妊娠结局风险[41]，但在孕早期仍应充分权衡治疗获益与潜在风险。

**图1:**
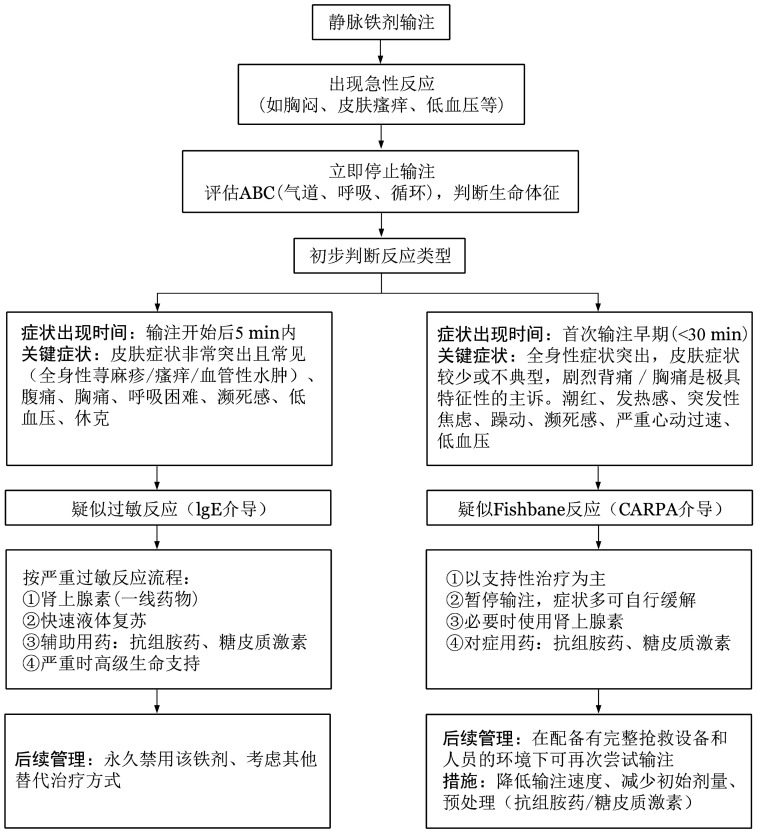
静脉铁剂输注相关过敏反应（IgE介导）和Fishbane反应（CARPA介导）的机制和处理整合示意图

（3）铁过载：铁过载可能损伤胰岛β细胞，增加妊娠糖尿病风险。对铁充足孕妇（血清铁蛋白≥50 µg/L），一般不建议预防性补铁；但对缺铁人群（血清铁蛋白<30 µg/L），补铁获益仍明显大于其理论风险。

（4）低磷血症：系统评价显示，FCM治疗组低磷血症（血磷<0.8 mmol/L）发生率可达92％，显著高于FDI组（4％）[42]。FCM通过激活成纤维细胞生长因子23（FGF23）信号通路，抑制肾小管磷重吸收，导致尿磷排泄增加[43]。低磷血症可能导致全身无力、疲劳和肌肉疼痛，还可能引发骨软化症和骨折。在妊娠期，低磷血症可能对胎儿的骨骼发育产生不利影响。2025年一项RCT表明，FCM相关低磷血症多为一过性（中位持续时间21 d），且未增加不良妊娠结局[44]。在加强血磷监测并避免重复给药的前提下，FCM仍可作为有效治疗选择。

四、国际与地区指南推荐

《铁缺乏症和缺铁性贫血诊治和预防的多学科专家共识（2022年版）》[45]推荐不能耐受口服铁剂或需要快速纠正铁缺乏的患者接受静脉铁剂治疗，不推荐在孕早期静脉补铁。

2023年《亚洲妇产科改善缺铁性贫血管理的专家共识》[46]推荐用于无法耐受口服铁剂或需要快速纠正IDA的患者。从孕中期开始使用静脉铁剂。

2024年美国专家共识[47]推荐在妊娠13周后使用静脉铁剂。对于有低磷血症风险的患者，尤其是需要重复输注者，应避免使用FCM，考虑使用其他静脉铁剂，如低分子量铁右旋糖酐铁或FDI。

五、结论

妊娠期IDA对母婴健康构成严重威胁，及时诊断和有效治疗至关重要。近年来多项RCT证实，静脉铁剂在纠正中重度IDA方面具有显著优势，且未增加不良妊娠结局的风险。新型静脉铁剂为妊娠期IDA治疗提供了新选择，但需平衡快速纠正贫血的临床需求与潜在代谢风险。未来仍需开展更多妊娠特异性高质量研究，重点关注母婴远期结局及不良反应风险预测，以进一步优化治疗决策，提高贫血纠正效率并改善妊娠结局。
